# Health-related quality of life in women with polycystic ovary syndrome attending to a tertiary hospital in Southeastern Spain: a case-control study

**DOI:** 10.1186/s12955-020-01484-z

**Published:** 2020-07-16

**Authors:** María L. Sánchez-Ferrer, Evdochia Adoamnei, María T. Prieto-Sánchez, Jaime Mendiola, Shiana Corbalán-Biyang, Miriam Moñino-García, Joaquín A. Palomar-Rodríguez, Alberto M. Torres-Cantero

**Affiliations:** 1 Department of Obstetrics & Gynecology, “Virgen de la Arrixaca” University Clinical Hospital, 30120 El Palmar (Murcia), Spain; 2 Institute for Biomedical Research of Murcia, IMIB-Arrixaca, 30120 El Palmar (Murcia), Spain; 3 Division of Preventive Medicine and Public Health, Department of Public Health Sciences, University of Murcia School of Medicine, 30100 Espinardo (Murcia), Spain; 4 Biomedical Research Centre Network for Epidemiology and Public Health (CIBERESP), 28029 Madrid, Spain; 5 Servicio de Planificación y Financiación Sanitaria, Consejería de Salud, Región de Murcia, 30001 Murcia, Spain; 6 Department of Preventive Medicine, “Virgen de la Arrixaca” University Clinical Hospital, 30120 El Palmar (Murcia), Spain

**Keywords:** Case-control study, Health-related quality of life, Polycystic ovary syndrome, SF-12v2

## Abstract

**Background:**

Polycystic ovary syndrome (PCOS) is a chronic condition with symptoms affecting many women at reproductive age and evaluating their health-related quality of Life (HRQoL) is an important issue. Moreover, differences in the HRQoL between women with different PCOS phenotypes have never been analyzed. Therefore, the aim of our study was to compare the HRQoL between women with PCOS -and its phenotypes- and controls attending to a tertiary hospital.

**Methods:**

A group of 117 women with PCOS and 153 controls were studied between 2014 and 2016. Controls were women without PCOS attending the gynecological outpatient clinic for routine examinations. Cases were women attending the same setting and diagnosed with PCOS. PCOS diagnose was performed following the Rotterdam Criteria and women were further classified by anovulatory or ovulatory phenotypic subtype. Women underwent physical and gynecological exams and completed health questionnaires including the Short Form-12v2. Eight scales and two component summary scores [Physical (PCS) and Mental (MCS), respectively] were calculated. Bivariate and multivariate analyses were performed to assess differences in HRQoL between women with PCOS and controls.

**Results:**

All women with PCOS and anovulatory PCOS presented lower score in PCS compared to controls [mean (95%CI): 53.7 (52.5–54.9) and 52.9 (51.5–54.4) vs. 55.8 (54.8–56.8); *p*-values< 0.01], as well as lower scores for five out of the eight scales (*p*-values < 0.05) after adjusting by age, body mass index, infertility, educational level and current occupation. No significant differences were observed for the MCS between women with or without PCOS or its phenotypic subtypes.

**Conclusions:**

HRQoL was significantly decreased in adult women with PCOS and its anovulatory phenotype compared to controls attending the outpatient clinic of a tertiary hospital. These results may have implications for the clinical practice and suggest the need for specific interventions in women with PCOS.

## Background

Polycystic ovary syndrome (PCOS) is one of the most common chronic endocrinopathies affecting between 5 and 10% of reproductive age women [27]. Clinical manifestations of this syndrome such as obesity, infertility, hirsutism, biochemical and hormonal disturbances has been widely described [4]. Yet, these symptoms are often related to a deterioration in the woman’s self-esteem and self-image and may affect their health-related quality of life (HRQoL), particularly in relationship with psychosocial domains [1, 6, 32].

HRQoL is a multidimensional concept widely used in medical research, but its usage in routine medical practice is increasing. It is defined as “individual’s perception of their own life in the context of their cultures and believes, and their personal goals and concerns” [3, 36]. Important areas such as physical health, psychological health, level of independence and social relationships are included in HRQoL evaluation. Over the past years, there has been a growing tendency to incorporate assessment of HRQoL in clinical studies and routine clinical management of PCOS.

Consequently, several investigations conducted over the world have shown associations between HRQoL and the presence of PCOS [4, 5, 12, 21, 25, 28, 32]. Women with PCOS may be at a higher risk of low HRQoL [7, 8, 16, 18, 37, 38]. However, several of the previous studies have focused on series of women with PCOS or evaluated the effect of an intervention (lifestyle or medical treatments) on HRQoL of women with PCOS [17, 34] without adequate control. Therefore, the interpretation and generalization from these studies is challenging, due to relatively small sample sizes, heterogeneities between study populations, tools evaluating HRQoL, or the inadequate control of confounding. The impact of potential confounders such as age, body mass index (BMI), educational level or even professional activity upon HRQoL in PCOS women is uncertain as they may not have been properly evaluated [2, 32]. Besides, there are differences in PCOS symptoms presented across geographical locations and between differing race or ethnic groups [11, 41].

Moreover, the Rotterdam ESHRE/ASRM definition recognizes four different phenotypes of this syndrome [27, 31], but whether there are differences in the HRQoL between the different phenotypes has never been analyzed. It is also important to know more about HRQoL in women suffering from this common problem in order to develop strategies and interventions to enhance their HRQoL.

Therefore, the goal of this work was to compare the HRQoL of adult women with PCOS -and its phenotypes- and controls. We hypothesize that women with PCOS, especially those with anovulatory phenotype, would show worse HRQoL compared to women without PCOS.

## Material and methods

### Study population

This was a case-control study conducted from September 2014 to May 2016 at the Department of Obstetrics and Gynecology of the University Clinical Hospital “Virgen de la Arrixaca” in the Murcia Region (southeastern Spain). The study conception and design have been previously described elsewhere [33]. Women were excluded if they: were < 18 and > 40 years old, had endocrine disorders (e.g. Cushing’s syndrome, congenital adrenal hyperplasia, androgen-secreting tumors, hyperprolactinemia and hyper- and hypothyroidism) or were taking any hormonal medication (including contraception) during the 3 months prior to the study; were pregnant or lactating; had been exposed to oncological treatment; or had genitourinary prolapse. For both groups, women with PCOS and controls, gynecologists recruited consecutive women attending the clinic (total *n* = 307), and more than 95 % of the approached women fulfilling the study criteria agreed to participate (*n* = 14 declined and *n* = 23 were excluded). Those that declined to participate was due to a lack of time for filling out questionnaires. Women with PCOS (*n* = 117) were women attending the gynecology unit of the hospital, and included newly diagnosed cases as well as prevalent ones. Diagnosis of PCOS was established following the Rotterdam criteria [31] which included a complete medical history with a modified Ferriman-Galwey (mF-G) score [19], transvaginal ultrasound (TVUS) and serum sexual hormones. Diagnosis of PCOS required completion of at least two of the following three criteria: (i) hyperandrogenism either biochemical (total testosterone level ≥ 2.6 nmol/l) or clinical (mF-G score ≥ 12) [1] with or without acne or androgenic alopecia; (ii) oligo- and/or anovulation (menstrual cycles > 35 days or amenorrhea > 3 months); (iii) polycystic ovarian morphology (POM) on TVUS (≥12 follicles measuring 2–9 mm in diameter, mean of both ovaries) [15]. Possible phenotypic subtypes were: phenotype A (oliganovulation + hyperandrogenism + polycystic ovary morphology); phenotype B (oliganovulation + hyperandrogenism); phenotype C (hyperandrogenism + polycystic ovary morphology); and phenotype D (oliganovulation + polycystic ovary morphology) [22]. Finally, A, B and D phenotypes were reclassified as “anovulatory phenotypes” (*n* = 84) and phenotype C as “ovulatory phenotype” (*n* = 33) and evaluated separately in the current study.

On the other hand, controls (*n* = 153) were women without PCOS (or other major gynecological conditions, e.g. endometriosis) attending the gynecological outpatient clinic for routine gynecological exams. The same procedures were performed in both women with PCOS and controls: anamnesis and questionnaires, physical examination including weight and height measured using a digital scale (Tanita SC 330-S, Amsterdam, The Netherlands). Uterine and ovarian morphology were evaluated with TVUS with Voluson E8® and 4–9 MHz transducer (General Electric Healthcare, USA) and blood draw between days 2.5 of the menstrual cycle. Written informed consent was obtained from all subjects. This study was approved by the Ethics Research Committee of the University of Murcia and the University Clinical Hospital (no. 770/2013, approved 3 October 2013).

### Health-related quality of life measurement

The Short Form (SF)-12v2 Health Survey is a validated shorter version of the SF-36 generic questionnaire that encompasses 12 items, evaluating physical and mental health from the participant’s point of view (4 weeks recall period) [20, 30, 39, 40]. The questionnaire generates eight scales: physical functioning, role physical, bodily pain, general health, role emotional, vitality, social functioning and mental health. All raw scale scores were converted to a 0–100 scale, with higher scores representing higher levels of HRQoL. Additionally, the subscales were also transformed to normative-based scores according to the SF-12v2 recommendations to give a mean of 50 and a standard deviation of 10, using a representative sample of the 1998 US general population [20, 30, 40]. This transformation allows to obtain two summary measures: Physical and Mental Component Summary (PCS and MCS, respectively) that may be directly compared with other scales and scores. As the mean score is set to 50, scores ≥50 or < 50 indicate better or worse physical or mental health than the 1998 US general population. Scores bounds are set at 48 (0.2 SD) for a small effect on HRQoL, 45 (0.5 SD) for a moderate influence and, f ≤ 42 (0.8 SD) for a large effect on HRQoL [13, 14].

### Statistical analyses

Descriptive statistics are presented using raw data. Continuous variables were compared using unpaired Student T tests, and categorical variables with chi-squared. Analysis of covariance was employed to calculate adjusted crude (0–100) and norm-based scales and component summaries differences between women with PCOS and controls. Multiple logistic regression was used to explore associations between women with PCOS and controls and norm-based scales and summary measures’ score with a cut-off of above/below 50 using odds ratios (OR) and 95% confidence intervals (95%CI). In both cases, several relevant covariates were considered (e.g. age, BMI, infertility problems, educational level, current employment, etc.) as potential confounders. When inclusion of a potential covariate resulted in a change in the β-coefficient of more than 10%, the variable was retained in the final models. These variables included factors previously related to PCOS in this or other studies, regardless of whether they had been previously described as predictors of PCOS. From previous publications [28, 29], we aimed to detect a difference of at least 3 points (with a standard deviation of about 7 points) in the global scores (PCS or MCS) between women with PCOS and controls. For an of alpha error of 0.05 and 80% statistical power to detect differences, a minimum of 90 women would be required in each group. All tests were two-tailed at 0.05 significance level. Analyses were conducted with IBM SPSS 25.0 (IBM Corporation, Armonk, New York, USA).

## Results

Overall, PCOS women were younger, had higher BMI, more infertility problems, and showed lower educational and current occupation level than controls. Regarding marital status and other lifestyle factors both groups were comparable (Table 1).

**Table 1:** Comparison of the general characteristics of women with PCOS and controls

Characteristics	Controls (*n* = 153)	All women with PCOS (*n* = 117)	*p*-value^c^
	Mean (SD)	Mean (SD)	
Age (years)	30.6 (5.9)	27.4 (5.0)	< 0.001
Height (m)	1.65 (0.05)	1.65 (0.06)	0.86
Weight (kg)	63.0 (11.3)	69.2 (17.3)	0.001
Body mass index (BMI) (kg/m^2^)	23.3 (4.3)	25.5 (5.9)	0.001
	N (%)		
Women with anovulatory PCOS	–	92 (71.8)	–
Women with ovulatory PCOS	–	34 (28.2)	–
Infertility problems	13 (8,6)	26 (22,2)	0.002
Alcohol consumption^a^	123 (83.1)	89 (78.1)	0.30
Tobacco consumption^b^	80 (53.0)	60 (52.6)	0.96
Educational level			
*Primary*	14 (9.2)	24 (20.4)	0.001
*Secondary*	38 (24.8)	39 (33.9)	
*University*	101 (66.0)	52 (45.2)	
Marital status			
*Other*	76 (49.7)	57 (48.7)	0.88
*Married*	77 (50.3)	60 (51.2)	
Current occupation			
*Unemployed*	22 (14.4)	24 (21.2)	0.04
*Studying*	34 (22.2)	35 (31.0)	
*Working*	97 (63.4)	54 (47.8)	

Data are presented as mean and standard deviation (SD) on Number (N) and percentage (%)

^a^Did you ever drink alcoholic beverages with a frequency of at least one a month?

^b^Have you ever smoked?

^c^T-student or chi-squared test

Table 2 shows the subscales (0–100) of the SF-12v2 questionnaire in women with PCOS and controls. In unadjusted analyses (data not shown), women with PCOS (vs. controls) scored significantly lower in the scales, except for physical functioning (*p* = 0.06), social functioning (*p* = 0.07) and mental health (*p* = 0.08). After adjustment, differences remained in four scales: role physical (*p* < 0.001), general health (*p* = 0.01), vitality (*p* = 0.04) and role emotional (*p* = 0.02). When women with ovulatory or anovulatory PCOS were compared to controls, in adjusted models, women with anovulatory PCOS scored lower in three scales: role physical (*p* < 0.001), vitality (*p* = 0.03) and role emotional (*p* = 0.02), while women with ovulatory PCOS scored lower in two scales [general health (*p* = 0.02) and mental health (*p* = 0.04)].

**Table 2:** Comparison of the eight health concept scales of the SF-12v2 questionnaire between all women with PCOS and phenotypic subtypes (women with ovulatory and anovulatory PCOS) and controls

Variables	Controls (*n* = 153)	All women with PCOS (*n* = 117)	*p*-value^a^	Women with ovulatory PCOS (*n* = 33)	*p*-value^a^	Women with anovulatory PCOS (*n* = 84)	*p*-value^a^
	Mean (SD)	Mean (SD)		Mean (SD)		Mean (SD)	
Physical Functioning	94.8 (15.6)	90.9 (19.6)	0.24	94.7 (13.6)	0.99	88.9 (21.4)	0.14
Role Physical	90.0 (16.2)	81.3 (20.4)	0.001	83.3 (22.5)	0.06	80.5 (19.6)	< 0.001
Bodily Pain	91.2 (17.3)	82.7 (23.1)	0.05	86.4 (25.1)	0.31	81.3 (22.3)	0.13
General Health	77.4 (18.6)	68.6 (20.9)	0.01	69.8 (19.7)	0.02	68.2 (21.4)	0.13
Vitality	65.5 (19.7)	59.4 (20.9)	0.04	64.4 (17.7)	0.90	57.4 (21.9)	0.03
Social Functioning	81.9 (20.9)	76.7 (23.6)	0.22	76.5 (25.7)	0.18	76.8 (22.9)	0.31
Role Emotional	80.6 (20.9)	72.1 (21.9)	0.02	72.0 (23.4)	0.08	72.2 (21.4)	0.02
Mental Health	65.1 (18.8)	61.0 (18.9)	0.28	57.6 (17.7)	0.04	62.4 (19.3)	0.72

Data are presented as mean and standard deviation (SD). Range values between 0 and 100, with higher scores representing higher levels of HRQoL

^a^Analysis of covariance (ANCOVA). Model adjusted by age, BMI, infertility problems, educational level and current occupation

**Fig. 1:**
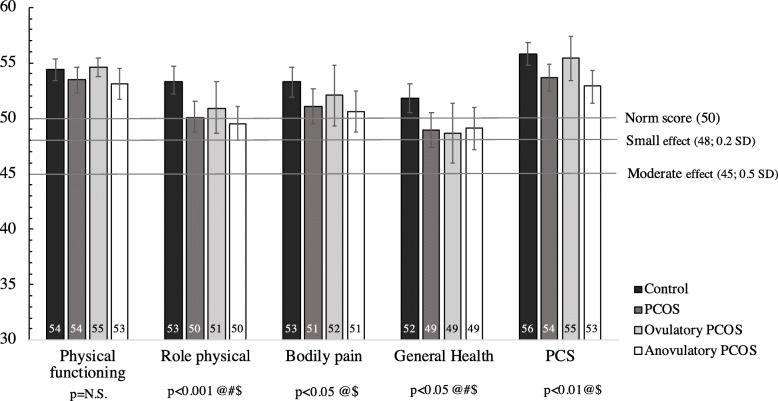
Adjusted means and 95%CI of the four scales and Physical Component Summary (PCS) of SF-12v2 questionnaire between all women with PCOS and its phenotypic subtypes (anovulatory and ovulatory) and controls. Model adjusted by age, BMI, infertility problems, educational level and current occupation. (@): significant differences between all women with PCOS and controls; (#) significant differences between ovulatory PCOS and controls; ($) significant differences between anovulatory PCOS and controls

**Fig. 2:**
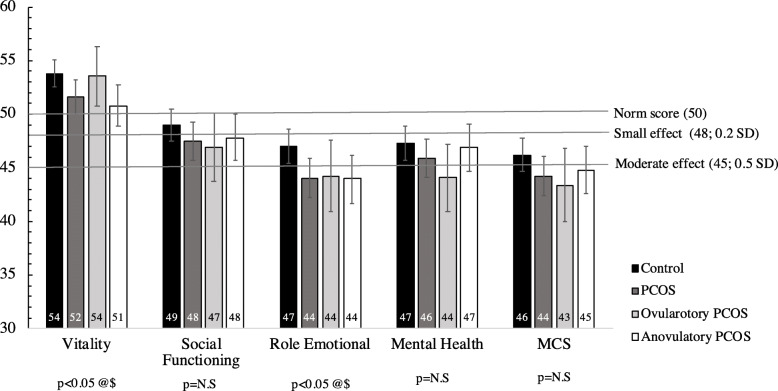
Adjusted means and 95%CI of the four scales and Mental Component Summary (MCS) of SF-12v2 questionnaire between all women with PCOS and its phenotypic subtypes (anovulatory and ovulatory) and controls. Model adjusted by age, BMI, infertility problems, educational level and current occupation. (@): significant differences between all women with PCOS and controls; ($) significant differences between anovulatory PCOS and controls

The assessment of the norm-based scales and summary measures’ scores of the SF-12v2 between all PCOS women -and phenotypic subtypes- and controls is shown in Table 3. Crude data are available in supplementary Table 1. In adjusted analyses, five scales showed significantly lower scores for all PCOS versus controls (role physical, bodily pain, general health, vitality and role emotional, having this last one the lowest score 44.0 vs. 47.0; *p* = 0.02). The PCS was also significantly lower in all PCOS women versus controls (53.7 vs. 55.8; *p* = 0.01). However, there was no significant difference in MCS, although both, all PCOS group and controls scored below 50 [44.2 vs. 46.2, *p* = 0.12]. When looking at phenotypic subtypes, anovulatory PCOS showed similar results compared to controls, but for ovulatory PCOS only general health presented significantly lower values compared to controls (*p* = 0.02). Adjusted means and 95%CI of the eight scales and the two component summary scores between all women with PCOS and its phenotypic subtypes (anovulatory and ovulatory) and controls can be seen on Figs. 1 and 2.

**Table 3:** Comparison of the norm-based scales and summary measures’ scores of SF-12v2 between all women with PCOS and phenotypic subtypes (women with ovulatory and anovulatory PCOS) and controls

Variables	Controls (*n* = 153)	All women with PCOS (*n* = 117)	*p*-value^a^	Women with ovulatory PCOS (*n* = 33)	*p*-value^a^	Women with anovulatory PCOS (*n* = 84)	*p*-value^a^
	Mean (95%CI)	Mean (95% CI)		Mean (95% CI)		Mean (95% CI)	
Physical Functioning	54.4 (53.4–55.3)	53.5 (52.3–54.6)	0.24	54.6 (53.8–55.4)	0.99	53.1 (51.7–54.5)	0.17
Role Physical	53.3 (52.2–54.5)	50.1 (48.8–51.5)	0.001	50.9 (48.7–53.3)	0.06	49.5 (47.9–51.0)	< 0.001
Bodily Pain	53.3 (51.9–54.6)	51.1 (49.5–52.7)	0.04	52.1 (49.3–54.8)	0.31	50.6 (48.7–52.4)	0.02
General Health	51.8 (50.5–53.1)	48.9 (47.4–50.5)	0.01	48.7 (46.0–51.4)	0.02	49.1 (47.2–51.0)	0.04
Vitality	53.8 (52.5–55.1)	51.6 (50.0–53.2)	0.04	53.6 (50.9–56.4)	0.90	50.8 (48.9–52.7)	0.02
Social Functioning	49.0 (47.5–50.5)	47.5 (45.7–49.3)	0.22	46.9 (43.7–50.1)	0.18	47.8 (45.6–49.9)	0.39
Role Emotional	47.0 (45.4–48.6)	44.0 (42.2–45.9)	0.02	44.2 (40.8–47.5)	0.11	44.0 (41.8–46.3)	0.03
Mental Health	47.3 (45.7–48.9)	45.9 (44.1–47.7)	0.28	44.1 (41.0–47.3)	0.06	46.9 (44.7–49.1)	0.82
PCS	55.8 (54.8–56.8)	53.7 (52.5–54.9)	0.01	55.4 (53.4–57.4)	0.53	52.9 (51.5–54.4)	0.002
MCS	46.2 (44.7–47.8)	44.2 (42.4–46.1)	0.12	43.4 (40.0–46.8)	0.11	44.8 (42.6–47.0)	0.32

Data are presented as mean and 95%CI. PCS: Physical Component Summary; MCS: Mental Component Summary. Norm-based scores in the US general population have a mean of 50 and a standard deviation of 10. Mean score is set to 50, therefore scores ≥50 or < 50 indicate better or worse physical or mental health than the mean US population, respectively

^a^Analysis of covariance (ANCOVA). Model adjusted by age, BMI, infertility problems, educational level and current occupation

Table 4 presents crude and adjusted OR and 95%CI for norm-based scales and summary measures’ scores of SF-12v2 between women with PCOS and controls. In full adjusted models, women with PCOS (compared to controls) were 2.13 times (95%CI:1.03–4.49) more likely to have PCS scores below 50. The strength of the relationship remained significant when the analysis was carried out for the PCS subscales (role physical, bodily pain or general health) with ORs ranging between 3.28 (95%CI: 1.81–5.96) for role physical to 1.76 (95%CI: 1.02–3.16) for bodily pain. When considering the MCS and its subscales, vitality, role emotional and mental health remained significant after multivariate adjustment. For instance, women with PCOS (compared to controls) were 1.99 (95%CI: 1.16–3.44), 2.11 (95%CI: 1.21–3.66) and 1.70 (95%CI: 1.02–2.99) times more likely to have vitality, role emotional or mental health scores below 50, respectively. Nonetheless, neither crude nor adjusted significant associations were found for social functioning or MCS.

**Table 4:** Crude and adjusted odds ratios (OR) and 95%CI for norm-based scales and summary measures’ scores of SF-12v2 between women with and without PCOS

Characteristics	Cut-offs	Controls (*n* = 153)	All women with PCOS (*n* = 117)	OR (95% CI) Crude Model	OR (95% CI) Adjusted Model^a^
Physical Functioning	≥50	133 (86.9)	90 (76.9)	Ref.	Ref.
	< 50	20 (13.1)	27 (23.1)	**2.00 (1.10–3.77)**	1.46 (0.67–3.17)
Role Physical	≥50	118 (77.1)	62 (53.0)	Ref.	Ref.
	< 50	35 (22.9)	55 (47.0)	**2.99 (1.77–5.05)**	**3.28 (1.81–5.96)**
Bodily Pain	≥50	115 (75.2)	64 (54.7)	Ref.	Ref.
	< 50	38 (24.8)	53 (45.3)	**2.51 (1.50–4.20)**	**1.76 (1.02–3.16)**
General Health	≥50	101 (67.3)	52 (44.4)	Ref.	Ref.
	< 50	50 (32.7)	65 (55.6)	**2.60 (1.57–4.23)**	**2.35 (1.32–4.17)**
PCS	≥50	135 (88.2)	89 (76.1)	Ref.	Ref.
	< 50	18 (11.8)	28 (23.9)	**2.36 (1.23–4.52)**	**2.13 (1.03–4.49)**
Vitality	≥50	92 (60.1)	49 (41.9)	Ref.	Ref.
	< 50	61 (39.9)	68 (58.1)	**2.09 (1.28–3.42)**	**1.99 (1.16–3.44)**
Social Functioning	≥50	78 (51.0)	48 (41.0)	Ref.	Ref.
	< 50	75 (49.0)	69 (59.0)	1.50 (0.92–2.43)	1.38 (0.80–2.40)
Role Emotional	≥50	82 (53.6)	42 (35.9)	Ref.	Ref.
	< 50	71 (46.4)	75 (64.1)	**2.06 (1.26–3.38)**	**2.11 (1.21–3.66)**
Mental Health	≥50	76 (49.7)	40 (34.2)	Ref.	Ref.
	< 50	77 (50.3)	77 (65.8)	**1.90 (1.16–3.12)**	**1.70 (1.02–2.99)**
MCS	≥50	60 (39.2)	36 (30.8)	Ref.	Ref.
	< 50	93 (60.8)	81 (69.2)	1.45 (0.87–2.42)	1.20 (0.68–2.14)

Data presented as number (N) and percentage (%). Norm-based scores in the US general population have a mean of 50 and a standard deviation of 10

Mean score is set to 50 (Cut-off), therefore scores ≥50 or < 50 indicate better or worse physical or mental health than the mean 1998 US population, respectively

*PCS* Physical Component Summary, *MCS* Mental Component Summary

Bold values are statistically significant (*p* < 0.05)

^a^Multiple logistic regression model adjusted by age and BMI, infertility problems, educational level and current occupation

Lastly, crude and adjusted OR and 95%CI for norm-based scales and summary measures’ scores of SF-12v2 between phenotypic subtypes (women with ovulatory or anovulatory PCOS) and controls are shown in Table 5. Final models showed that women with anovulatory PCOS were 2.65 (95%CI: 1.14–6.20) times more likely to present worse PCS (< 50) and all their subscales but physical functioning. Moreover, women anovulatory PCOS were 2.35 (95%IC:1.23–4.48) times more likely to have role emotional scores below 50. On the other hand, for women with ovulatory PCOS only, the subscales general health and mental health reached a significant association, showing that these women were 2.42 (95%IC:1.03–5.78) and 2.98 times (95%IC:1.20–7.37), respectively, more likely to have scores below 50 compared to controls.

**Table 5:** Crude and adjusted odds ratios (OR) for norm-based scales and summary measures’ scores of SF-12v2 between women with phenotypic subtypes of PCOS (ovulatory and anovulatory PCOS) and controls

Characteristics	Cut-offs	Controls (*n* = 153)	Women with ovulatory PCOS (*n* = 33)	OR (95% CI) Crude Model	OR (95% CI) Adjusted Model^a^	Women with anovulatory PCOS (*n* = 84)	OR (95% CI) Crude Model	OR (95% CI) Adjusted Model^a^
		n (%)	n (%)			n (%)		
Physical Functioning	≥50	133 (86.9)	28 (84.8)	Ref.	Ref.	62 (73.8)	Ref.	Ref.
	< 50	20 (13.1)	5 (15.2)	1.18 (0.44–3.43)	1.10 (0.30–3.94)	22 (26.2)	**2.36 (1.20–4.64)**	1.65 (0.68–3.98)
Role Physical	≥50	118 (77.1)	22 (66.7)	Ref.	Ref.	40 (47.6)	Ref.	Ref.
	< 50	35 (22.9)	11 (33.3)	1.69 (0.74–3.81)	1.61 (0.64–4.03)	44 (55.7)	**3.71 (2.10–6.56)**	**4.76 (2.41–9.39)**
Bodily Pain	≥50	115 (75.2)	24 (72.7)	Ref.	Ref.	40 (47.6)	Ref.	Ref.
	< 50	38 (24.8)	9 (27.3)	1.14 (0.49–2.65)	1.20 (0.46–3.13)	44 (52.4)	**3.33 (1.89–5.85)**	**2.34 (1.22–4.52)**
General Health	≥50	103 (67.3)	16 (48.5)	Ref.	Ref.	36 (42.9)	Ref.	Ref.
	< 50	50 (32.7)	17 (51.5)	**2.19 (1.02–4.69)**	**2.42 (1.03–5.78)**	48 (57.1)	**2.75 (1.59–4.75)**	**2.42 (1.25–4.67)**
PCS	≥50	135 (88.2)	27 (81.8)	Ref.	Ref.	62 (73.8)	Ref.	Ref.
	< 50	18 (11.8)	6 (18.2)	1.67 (0.61–4.59)	1.38 (0.41–4.66)	22 (26.2)	**2.66 (1.33–5.13)**	**2.65 (1.14–6.20)**
Vitality	≥50	92 (60.1)	17 (51.5)	Ref.	Ref.	32 (38.1)	Ref.	Ref.
	< 50	61 (39.9)	16 (48.5)	1.42 (0.67–3.02)	1.50 (0.66–3.43)	52 (61.9)	**2.45 (1.42–4.23)**	**2.11 (1.13–3.96)**
Social Functioning	≥50	78 (51.0)	15 (45.5)	Ref.	Ref.	33 (39.3)	Ref.	Ref.
	< 50	75 (49.0)	18 (54.5)	1.23 (0.59–2.66)	1.22 (0.53–2.81)	51 (60.7)	1.67 (0.94–2.76)	1.52 (0.81–2.87)
Role Emotional	≥50	82 (53.6)	13 (39.4)	Ref.	Ref.	29 (34.5)	Ref.	Ref.
	< 50	71 (46.4)	20 (60.6)	1.78 (0.82–3.82)	1.89 (0.82–4.35)	55 (65.5)	**2.19 (1.26–3.80)**	**2.35 (1.23–4.48)**
Mental Health	≥50	76 (49.7)	9 (27.3)	Ref.	Ref.	31 (36.9)	Ref.	Ref.
	< 50	77 (50.3)	24 (72.7)	**2.63 (1.15–6.03)**	**2.98 (1.20–7.37)**	53 (63.1)	1.69 (0.98–2.91)	1.32 (0.70–2.48)
MCS	≥50	60 (39.2)	10 (30.3)	Ref.	Ref.	26 (31.0)	Ref.	Ref.
	< 50	93 (60.8)	23 (69.7)	1.48 (0.66–3.33)	1.50 (0.61–3.67)	58 (69.0)	1.44 (0.82–2.53)	1.10 (0.56–2.13)

Data presented as number (N) and percentage (%). Norm-based scores in the US general population have a mean of 50 and a standard deviation of 10

Mean score is set to 50 (Cut-off), therefore scores ≥50 or < 50 indicate better or worse physical or mental health than the mean US population, respectively

*PCS* Physical Component Summary, *MCS* Mental Component Summary

Bold values are statistically significant (*p* < 0.05)

^a^Multiple logistic regression model adjusted by age and BMI, infertility problems, educational level and current occupation

## Discussion

HRQoL of women with PCOS -and especially, anovulatory PCOS- was significantly decreased compared to controls. Overall, these results suggest that PCOS may play an important role and have a potential effect on HRQoL in these Mediterranean women. To the best of our knowledge, this is the first study evaluating phenotypic subtypes of PCOS in relation to HRQoL.

It is known that PCOS have a significant negative impact on women’s HRQoL. Several authors have reported that PCOS women show worse HRQoL compared to women without the disorder [5, 12, 21, 25]. Moreover, problems with sexual satisfaction and increased psychological disturbances have been reported as well [21]. A recent meta-analysis concluded that having PCOS significantly reduced HRQoL in adolescent girls [24].

In our study, patients with PCOS had significantly lower scores in several subscales and in the PCS, which is somewhat consistent with the previously published literature on the matter [10, 28, 29]. Benson et al. [10] carried out a nation-wide survey in Germany using the SF-12 scale in a cohort of women with PCOS and observed that women with PCOS were at higher risk of common psychiatric disorders such as anxiety, depression or both, and these disorders were related to lower HRQoL. Other authors reported significantly lower scores in the short form 36 (SF-36) questionnaire in women with PCOS compared to controls [28] in both PCS and MCS [35]. Lastly, Panico et al. [29] reported worse HRQoL in women with PCOS compared to controls in the subscales of vitality and role emotional, although no differences for body pain were found, using the SF-36 questionnaire. However, they also reported significant differences regarding mental health and social and physical functioning, which were not found in our study population. On the other hand, changes in role physical and general health found in our study population were not observed by Panico et al. [29]. The discrepancies between those findings and our study might be attributed to differences in the reported results, since in those studies only crude results are shown, and no further adjustments are made by potential confounders (e.g. age, BMI, etc.). An alternative explanation, though unlikely explanation that would require further study, might be that there are true specific differences in the HRQoL of PCOS women in Southern Spain.

In a meta-analysis of Li et al. [25], five studies using SF-36 were included to evaluate the impact of PCOS on specific HRQoL domains. They concluded that women with PCOS obtained lower scores in all the analysed domains compared to controls and that the most affected one was the emotional role. These findings are in agreement with ours, since the emotional role domain was one of most affected one in both, women with PCOS and controls.

It is important to bear in mind that our participants were enrolled in a tertiary care center, therefore results may vary from other kind of populations (secondary care, patient’s associations, etc.). Nonetheless, controls from our sample also presented relatively low MCS scores (mean = 46.2), which is lower than previous studies [Benson et al. (mean MSC = 51.3); or Ozcan Dag et al. (mean MSC 62.6)]. This might be the reason why no difference was observed between women with PCOS and controls for MCS in our study population. Moreover, women with anovulatory PCOS are mainly characterized by oliganovulation and hyperandrogenism. Both features are quite related to infertility and self-esteem or self-concept issues, therefore we hypothesize this might be one of the main reasons we observed more significant differences in HRQoL for women with anovulatory PCOS instead of ovulatory PCOS.

There are studies suggesting that interventions focusing on changes in lifestyles or medical treatments [17, 34] might help to improve HRQoL in women with PCOS. According to our results, these suggested interventions might also be appropriate when it comes to phenotypic subtypes -mainly anovulatory women- but the current evidence is, to our knowledge, very limited and further interventional research regarding improvement of HRQoL in PCOS phenotypes is warranted.

The Polycystic Ovary Syndrome Questionnaire (PCOSQ) [23] and SF-36 are the most frequently instruments used for the assessment of HRQoL in PCOS women [9]. However, PCOSQ has not been validated in Spanish and is a specific questionnaire for PCOS which was not considered appropriate for a case- control study. We chose to use the SF-12v2 in the present study. It is shorter than the SF-36 and offers a measurement of health with a multidimensional nature, it is easy handling and worldwide used. Moreover, it has been validated and is extensively used in Spanish studies [26].

This research is not without limitations. Selection and measurement bias has always to be considered in case-control designs. Nonetheless, controls were women attending the public hospital in the same period and they stem from the same population from which women with PCOS emerged. Misclassification of disease status or the exposure (HRQoL) may have occurred, but, if present, it would contribute to underestimate the true magnitude of associations. Lastly, from the four phenotypes in the Rotterdam criteria we chose to dichotomize into two phenotypes (ovulatory and anovulatory) due to small numbers in the groups and that might have affected the results. However, this dichotomization has been previously used before supporting our current approach [42].

## Conclusions

Our results support the hypothesis that HRQoL is significantly decreased in adult women with PCOS and its anovulatory phenotype compared to controls. PCOS is a chronic and highly prevalent disorder in reproductive age women, therefore it may be important to assess HRQoL as a way of measuring their progression alongside the treatment in a follow-up. If confirmed, these results may have important implications for prevention, clinical practice and intervention in women with this condition, especially those with the anovulatory phenotype who seem to be the most affected ones in terms of HRQoL. These women could benefit from the implementation of medical and psychological actions to improve their quality of life.

## Supplementary information

**Additional file 1: Table S1.** Comparison of the norm-based scales and summary measures’ scores of SF-12v2 between women with PCOS and its phenotypes and controls (crude data).

## Data Availability

The datasets used and/or analysed during the current study are available from the corresponding author on reasonable request.

## Abbreviations

BMI: Body mass index
HRQoL: Health-related quality of Life
MCS: Mental component summary
PCS: Physical component summary
PCOS: Polycystic ovary syndrome
