# Supplementary material for: Health-related quality of life in women with polycystic ovary syndrome attending to a tertiary hospital in Southeastern Spain: a case-control study
Source: Health Qual Life Outcomes. 2020 Jul 16;18:232. doi: 10.1186/s12955-020-01484-z (PMC7364602; doi:10.1186/s12955-020-01484-z)
Supplement: Supplementary file 1 — Additional file 1: Table S1. Comparison of the norm-based scales and summary measures’ scores of SF-12v2 between women with PCOS and its phenotypes and controls (crude data). [file 12955_2020_1484_MOESM1_ESM.docx]

Supplementary Table 1. Comparison of the norm-based scales and summary measures’ scores of SF-12v2 between women with PCOS and its phenotypes and controls (crude data).

| Variables | Controls (n=153) | All women with PCOS (n=117) | p-values^a^ | Women with ovulatory PCOS (n=33) | p-values^a^ | Women with anovulatory_PCOS (n=84) | p-values^a^ |
| --- | --- | --- | --- | --- | --- | --- | --- |
|  | Mean (SD) | Mean (SD) |  | Mean (SD) |  | Mean (SD) |  |
| Physical Functioning | 54.7 (5.4) | 53.2 (6.7) | 0.06 | 54.6 (4.7) | 0.98 | 52.7 (7.4) | 0.02 |
| Role Physical | 53.2 (6.7) | 50.3 (7.5) | <0.001 | 51.0 (7.3) | 0.05 | 49.9 (7.2) | <0.001 |
| Bodily Pain | 53.8 (7.1) | 50.4 (9.4) | 0.001 | 51.9 (10.2) | 0.19 | 49.8 (9.1) | <0.001 |
| General Health | 52.3 (8.0) | 48.5 (9.0) | <0.001 | 49.0 (8.5) | 0.04 | 48.3 (9.2) | 0.001 |
| PCS | 56.1 (5.9) | 53.5 (6.6) | 0.001 | 55.4 (5.5) | 0.51 | 52.7 (6.9) | <0.001 |
| Vitality | 54.0 (7.9) | 51.5 (8.4) | 0.01 | 53.5 (7.1) | 0.76 | 50.7 (8.8) | 0.004 |
| Social Functioning | 49.2 (9.1) | 47.2 (9.5) | 0.10 | 47.1 (10.4) | 0.23 | 47.2 (9.2) | 0.10 |
| Role Emotional | 47.4 (9.3) | 43.6 (9.8) | 0.001 | 43.5 (10.5) | 0.04 | 43.6 (9.6) | 0.003 |
| Mental Health | 47.5 (9.2) | 45.5 (9.2) | 0.10 | 43.6 (8.6) | 0.03 | 46.2 (9.4) | 0.29 |
| MCS | 46.5 (9.8) | 43.9 (9.1) | 0.02 | 42.9 (9.5) | 0.06 | 44.2 (9.0) | 0.08 |

Data are presents as mean and standard deviation (SD). PCS: Physical Component Summary; MCS: Mental Component Summary. Norm-based scores in the US general population have a mean of 50 and a standard deviation of 10. Mean score is set to 50, therefore scores ≥50 or <50 indicate better or worse physical or mental health than the mean US population, respectively.

^a^ T-student test was use.
